# Peroxymonosulfate Activation by Bi_2_WO_6_/BiOCl Heterojunction Nanocomposites under Visible Light for Bisphenol A Degradation

**DOI:** 10.3390/nano11113130

**Published:** 2021-11-20

**Authors:** Yongkui Huang, Xiangyang Yin, Pei He, Shuangwu Kou, Xiaoting Zhang, Lei Wang, Peili Lu

**Affiliations:** 1 Key Laboratory of Shale Gas Exploration, Ministry of Natural Resources, Chongqing Institute of Geology and Mineral Resources, Chongqing 401120, China; hepei131180@163.com; 2 State Key Laboratory of Coal Mine Disaster Dynamics and Control, College of Environment and Ecology, Chongqing University, Chongqing 400044, China; hnsqyxy@163.com (X.Y.); kousw@hotmail.com (S.K.); szxyzxt@163.com (X.Z.); wanglei137319@cqu.edu.cn (L.W.)

**Keywords:** peroxymonosulfate, BiOCl, Bi_2_WO_6_, photocatalysis, organic contaminant

## Abstract

The combination of peroxymonosulfate (PMS) activation and photocatalysis has proven to be effective for organic contaminants treatment. However, the construction of an efficient catalytic material is an important challenge. Herein, novel Bi_2_WO_6_/BiOCl heterojunction nanocomposites were successfully designed and fabricated using a facile and effective strategy for bisphenol A (BPA) photodegradation with PMS activation. The well-designed heterojunction with improvement of the contact area and interface microstructure was obtained through in situ growth of the Bi_2_WO_6_ on the surface of BiOCl. The Bi_2_WO_6_/BiOCl nanocomposites exhibit excellent catalytic performance in PMS activation for BPA degradation under visible light irradiation. A possible photocatalytic reaction mechanism was systematically revealed. The excellent catalytic performance is mainly attributed to the strong interaction between Bi_2_WO_6_ and BiOCl, resulting in an enhanced photoabsorption and a more efficient interfacial charge separation and transfer. This paper provides a novel strategy to design efficient catalytic materials for organic contaminants remediation with PMS activation.

## 1. Introduction

Peroxymonosulfate (PMS) activation processes have been recognized as promising methods for practical environmental remediation of emerging recalcitrant contaminants [1,2,3]. The SO_4_^•−^ with superior oxidation ability and selectivity can efficiently degrade organic contaminants [1]. The metal ions, composites, and carbonaceous catalyst are frequently exploited to activate PMS [4,5]. However, concerns have been raised about the inevitable leaching of toxic metals and secondary contamination. The combination of PMS activation and photocatalyst has become a research focus because of their synergistic effect in the mineralization of organic contaminants [6,7]. Unfortunately, the main challenge faced by this technology is the lack of a highly efficient catalytic material [8,9]. Therefore, exploring efficient and environmentally benign materials for PMS activation is highly desirable.

In recent decades, Bi-based materials have attracted increasing interest in the areas of photocatalytic applications [10]. In particular, bismuth oxychloride (BiOCl) has attracted significant interest due to its unique crystallographic structure, which can establish an internal electric field for interlayer charge transfer [11,12]. However, the catalytic performance of BiOCl is restricted by the poor photogenerated charge separation ability and inactiveness of visible light utilization, which reduces the overall efficiency [13,14]. Therefore, various strategies, such as surface modification, defect engineering, morphology control, and heterojunction construction, have been widely adopted to tailor the photocatalytic activity of BiOCl [10,13]. The photocatalytic activity of BiOCl has been improved by these modification methods to some extent, but BiOCl-based catalysts are still unsatisfactory for environmental applications.

The light response and charge separation are two important factors to improve photocatalytic activity of semiconductors [15,16]. The construction of heterojunction composites has been studied in order to enhance the light absorption, the charge separation efficiency, and the lifetime of carriers [10,17]. Up to now, various BiOCl-based composites have been developed on the basis of this fundamental mechanism, such as BiOCl/g-C_3_N_4_ [14], BiOCl/Bi_24_O_31_Cl_10_ [18], and NiO/BiOCl [19]. It is well known that the heterojunction effect is established by an electronic coupling effect produced from the interfacial interactions of two components to enhance light-responsive capability and expedite interfacial charge carrier mobility [10,20]. However, the conventional large-sized materials could not construct perfect heterojunction interfaces. Recently, interface engineering has often been applied to explore the design of heterostructures [21,22]. The preparation process of a specific heterojunction is expensive and complicated in the context of mass production [23,24,25]. Therefore, exploring a facile strategy to create efficient heterojunction photocatalysts is highly challenging and appealing.

Herein, we have successfully designed a facile route to build Bi_2_WO_6_/BiOCl heterojunctions for organic pollutants degradation with PMS activation. The well-designed heterojunction with improvement of the contact area and interface microstructure was obtained through the growth of Bi_2_WO_6_ on the surface of BiOCl nanoplates, which showed extremely high levels of photocatalytic activity and stability in bisphenol A (BPA) degradation with PMS activation. Furthermore, the possible reaction mechanism was revealed by radical trapping experiments, charge transportation, and band structures. These findings provide an efficient approach to design novel heterojunction materials for organic contaminants remediation.

## 2. Experimental Section

### 2.1. Preparation of Materials

The BiOCl nanoplate was constructed via a hydrothermal method. In general, 2 mmol of cetyltrimethylammonium chloride (CTAC) was added to 20 mL of deionized water, and added into 2 mmol of Bi(NO_3_)_3_•6H_2_O aqueous solution (40 mL) drop by drop. After 2 h of stirring, the resulting solution was poured into a 100 mL Teflon-lined autoclave and treated at 120 °C for 12 h. Then, the samples were washed several times with ultrapure water.

The Bi_2_WO_6_/BiOCl heterojunction was constructed by in situ growth strategy. Briefly, 0.26 g of BiOCl nanoplate dispersed ultrasonically in 60 mL of water for 1 h. A certain amount of Na_2_WO_4_·2H_2_O was added into the suspension. After being stirred for another 2 h, the suspension were poured into a 100 mL Teflon-lined autoclave and treated at 120 °C for 12 h. The precipitation was obtained after being washed with ultrapure water.

A series of Bi_2_WO_6_/BiOCl heterojunctions with different weight percentages of Bi_2_WO_6_ (13%, 25%, 47%, and 75%) were prepared under the identical experimental conditions and denoted as BC-1, BC-2, BC-3, and BC-4, respectively. Pure Bi_2_WO_6_ was also fabricated through the same process with Bi(NO_3_)_3_•6H_2_O instead of BiOCl.

### 2.2. Materials Characterization

The X-ray powder diffraction (XRD) patterns were carried out by PANalytical powder X-ray diffractometer. Raman spectra of the samples were obtained on LabRAM HR Raman spectroscopy (HORIBA, Longjumeau, France). Field-emission scanning electron microscopy (FE-SEM) images were recorded by a JSM-7800F microscope (Tokyo, Japan). The transmission electron microscope (TEM) and EDX spectrometry were collected with a Talos F200S microscope (Thermo Fisher Scientific, Waltham, MA, USA). X-ray photoelectron spectroscopy (XPS) were collected with a photoelectron spectroscope (Thermo Fisher Escalab 250Xi, Waltham, MA, USA). The UV-vis diffuse reflectance spectroscopy spectra (UV-vis DRS) were obtained with a Shimadzu UV-3600 spectrophotometer (Shimadzu, Kyoto, Japan). The N_2_ adsorption–desorption measurement was measured using ASAP 2020 (Micromeritics Instrument Corp, Norcross, GA, USA). The time-resolved fluorescence decay spectra and photoluminescence (TRPL) spectra was tested by FLS-920 fluorescence spectrometer (Edinburgh Instruments, Edinburgh, UK). The electrochemical impedance spectroscopy (EIS), photocurrent response, and Mott–Schottky curves were tested on a CHI-660E electrochemical workstation (CH Instruments, Shanghai, China) with Ag/AgCl, Pt plate, and 0.5 M Na_2_SO_4_ aqueous solution as reference electrode, counter electrode, and electrolyte, respectively.

### 2.3. Activity Evaluation

The activity evaluation of samples was conducted through the photodegradation of BPA with PMS activation under irradiation by 300 W Xe lamp with a 400 nm cut-off filter. Briefly, 50 mg of the sample was dispersed into a BPA solution (50 mL, 10 mg/L) under stirring for 30 min in the dark. After adding a specific amount of PMS, the lamp was triggered. The concentration of PMS was 1 mmol/L. The BPA residual concentration was analyzed using high-performance liquid chromatography (Agilent 1260, Santa Clara, CA, USA) with a C-18 column and UV wavelength at 276 nm. The mobile phase was 70% methanol and 30% water, the flow rate was 1.0 mL/min.

## 3. Results and Discussion

### 3.1. Characterizations of Catalysts

**Figure 1:**
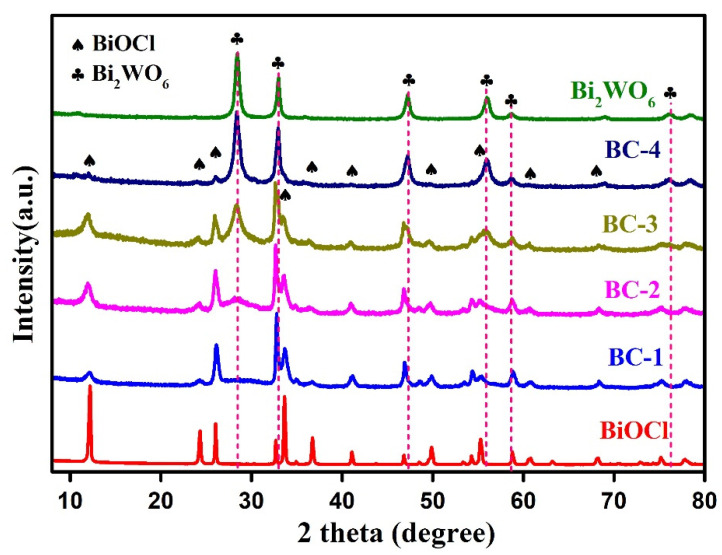
XRD patterns of BiOCl, Bi_2_WO_6_, and Bi_2_WO_6_/BiOCl nanocomposites.

Figure 1 presents the XRD patterns of the samples. The peaks of pure BiOCl are well assigned to the tetragonal phase (JCPDS no. 06-0249), implying the high purity of BiOCl nanoplates [13,14]. The peaks of Bi_2_WO_6_ are assigned to the orthorhombic phase (JCPDS No. 39-0256) [26,27]. The main peaks of the nanocomposites could be well assigned to the tetragonal BiOCl. The diffraction peaks of Bi_2_WO_6_ intensified with increasing mass content of Bi_2_WO_6_, evidencing the formation of the Bi_2_WO_6_ in the nanocomposites. These results confirm that Bi_2_WO_6_/BiOCl nanocomposites were successfully prepared.

**Figure 2:**
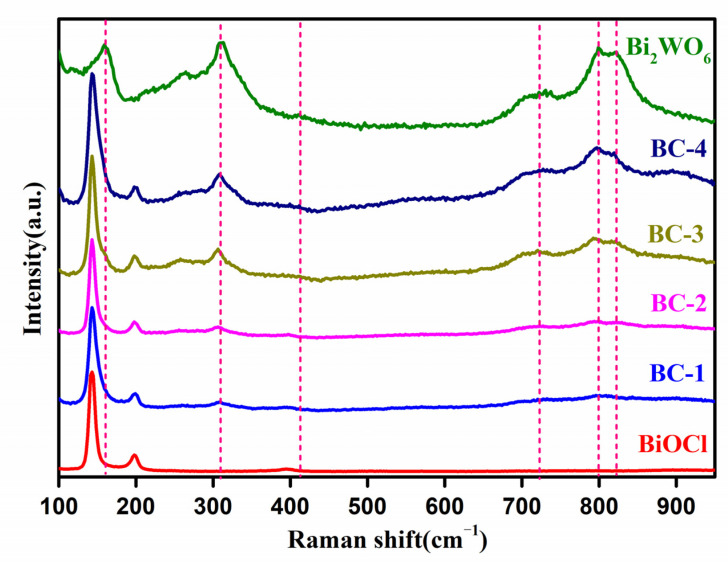
Raman spectra of BiOCl, Bi_2_WO_6_, and Bi_2_WO_6_/BiOCl nanocomposites.

Figure 2 shows the Raman spectra of samples. The pure BiOCl nanoplates exhibited the two peaks at 144 and 199.8 cm^−1^ due to the Bi-Cl stretching mode in BiOCl [12,28]. For pure Bi_2_WO_6_, the strong peak at 309 cm^−1^ is associated with the translational modes of Bi^3+^ and WO_6_^6−^. A weak peak centered at 410 cm^−1^ is assigned to WO_6_ bending (Eu) modes [29,30]. The broad band at 721 cm^−1^ is mainly assigned to the asymmetric stretching vibration of W plane and O [23,31]. The peaks at 798 and 824 cm^−1^ are associated with antisymmetric and symmetric Ag modes of O-W-O, respectively [23,29,30]. The characteristic peaks of Bi_2_WO_6_ and BiOCl are both shown in the spectra of the nanocomposites. Furthermore, the peaks intensity of Bi_2_WO_6_ gradually increased with increasing content of Bi_2_WO_6_, evidencing the presence of Bi_2_WO_6_ in the nanocomposites. The results explicitly confirm that the coexistence of BiOCl nanoplates and Bi_2_WO_6_ in the nanocomposites.

**Figure 3:**
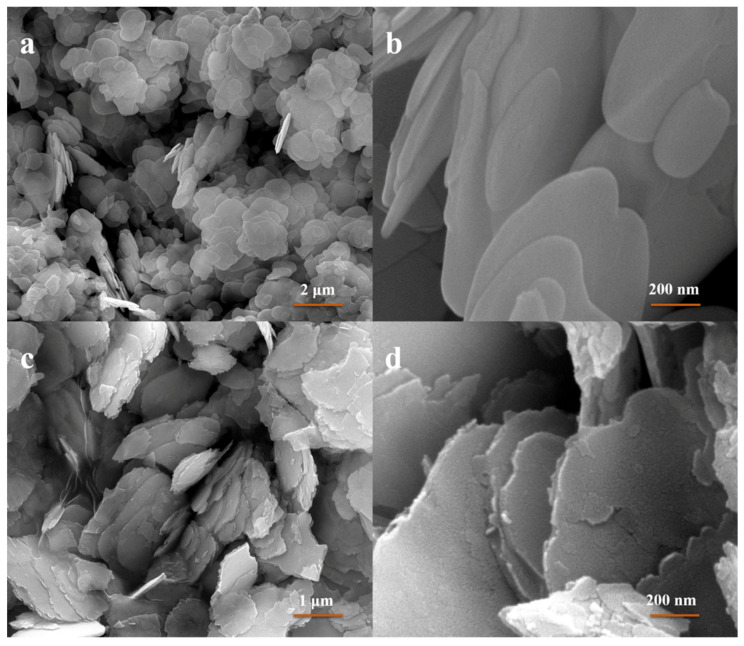
FE-SEM images of pure BiOCl nanoplates (**a**,**b**) and BC-3 nanocomposite(**c**,**d**).

The morphology of the samples was observed using FE-SEM. As shown in Figure 3a, pure BiOCl nanoplates displayed an ultrathin plate structure with a size ranging from 500 nm~2 μm. The high-magnification SEM image (Figure 3b) reveals the plate had a smooth surface with thickness of about 20 nm. Furthermore, BC-3 nanocomposite preserved a wrinkled plate structure assembled from ultrathin nanosheets (Figure 3c). Figure 3d clearly shows that the surfaces of the plates became rough, which can be ascribed to the growth of Bi_2_WO_6_ on the surface of BiOCl. This result reveals that Bi_2_WO_6_ has been successfully grown on the surface of BiOCl.

**Figure 4:**
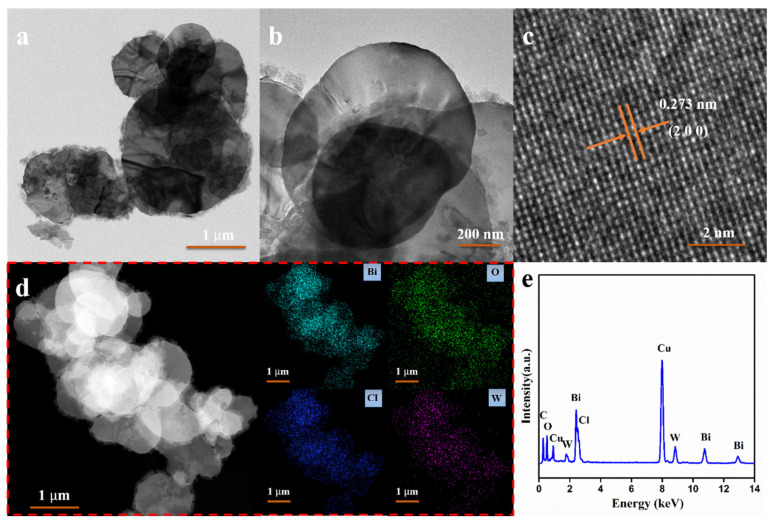
TEM (**a**,**b**) and HRTEM (**c**) images, elemental mapping images (**d**) and EDS spectrum (**e**) of BC-3 nanocomposites.

The TEM and HRTEM images of the BC-3 nanocomposite are shown in Figure 4. An ultrathin plate morphology of the BC-3 nanocomposite can be clearly observed from Figure 4a,b. The HRTEM image (Figure 4c) clearly reveals the clear lattice fringes. The lattice spacing was confirmed to be 0.272 nm, corresponding to (2 0 0) plane of orthorhombic Bi_2_WO_6_ [32,33]. Moreover, the elemental mapping (Figure 4d) and EDX spectrum (Figure 4e) present the uniform distribution of Bi, O, Cl, and W elements in the nanocomposite. Those observations explicitly demonstrate that the in situ growth method resulted in a strong interfacial contact and a large contact area between the Bi_2_WO_6_ and BiOCl.

**Figure 5:**
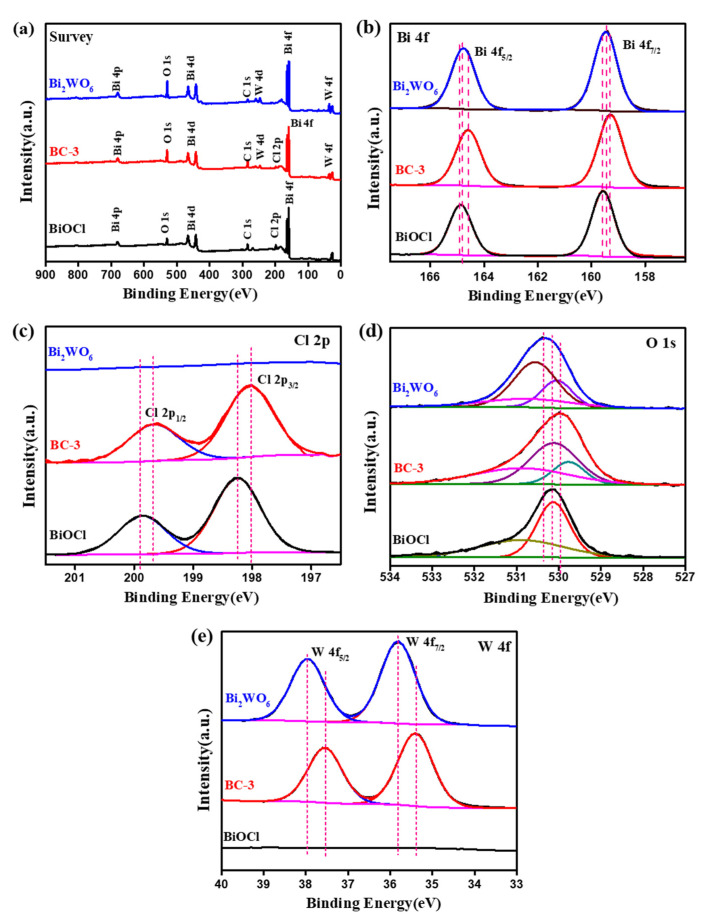
XPS spectra of pure BiOCl, Bi_2_WO_6_, and BC-3: survey spectrum (**a**), Bi4f (**b**), Cl 2p (**c**), O 1s (**d**), and W 4f (**e**).

The XPS spectra of the samples are illustrated in Figure 5. As exhibited in Figure 5a, the survey scan spectra indicate that BC-3 nanocomposites are mainly composed of Bi, O, Cl, and W elements. From the Bi 4f spectra of pure BiOCl (Figure 5b), the peaks of Bi 4f_5/2_ and Bi 4f_7/2_ at 164.9 and 159.6 eV indicate bismuth existed in the form of Bi^3+^, respectively [13,34]. Notably, the Bi 4f_7/2_ and Bi 4f_5/2_ in the BC-3 nanocomposites were located at about 164.6 and159.3 eV, respectively [35,36]. These shifts can be attributed to the chemical bonding actions among the components. The Cl 2p_3/2_ and Cl 2p_1/2_ in BiOCl (Figure 5c) were located at 198.2 and 199.9 eV, which are characteristic positions of the Cl^−^ [11,28]. These peaks changed to 198.0 and 199.7 eV after the construction of the Bi_2_WO_6_/BiOCl heterojunction, respectively. The XPS spectrum of O 1s in BiOCl (Figure 5d) displayed peaks at 531.0 and 530.1 eV, attributed to the lattice and surface oxygen [35,37,38]. Additionally, the O 1s spectrum of BC-3 nanocomposite was divided into three peaks at 531.0, 530.1, and 529.8 eV, corresponding to Bi-O, W-O, and hydroxyl groups, respectively [30,34]. From W 4f spectrum of BC-3 nanocomposite (Figure 5e), the peaks of W 4f at 37.5 and 35.4 eV are characteristic of the W^6+^ cations in Bi_2_WO_6_ [39,40]. The results indicate a strong interfacial contact between BiOCl and Bi_2_WO_6_ in Bi_2_WO_6_/BiOCl nanocomposites.

**Figure 6:**
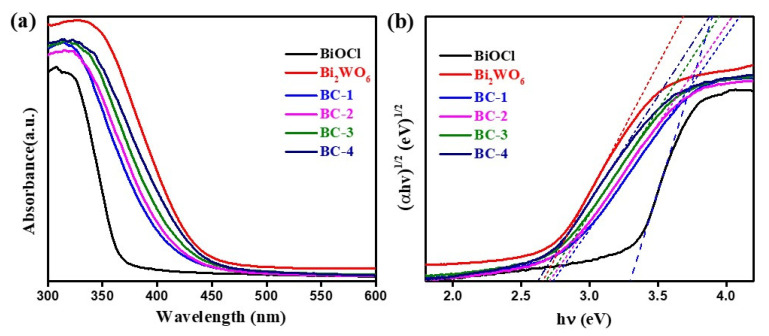
UV-vis DRS (**a**) and Tauc plots (**b**) of pure BiOCl, Bi_2_WO_6_, and Bi_2_WO_6_/BiOCl nanocomposites.

The UV-vis DRS of the samples is displayed in Figure 6a. The pure BiOCl exhibited a strong absorption in UV light regions and an absorption edge of around 370 nm, consistent with the literature reports [28,41]. Obviously, pure Bi_2_WO_6_ has an absorption edge of around 460 nm [42,43]. The Bi_2_WO_6_/BiOCl nanocomposites presented clear visible light absorption. Moreover, the absorption intensity gradually enhanced with increasing Bi_2_WO_6_ content. Furthermore, the band gap energy of the samples was studied (Figure 6b) [18,30]. The band gap energy values of pure BiOCl, Bi_2_WO_6_, BC-1, BC-2, BC-3, and BC-4 were 3.31, 2.66, 2.74, 2.68, and 2.62 eV, respectively, which demonstrates that the band gap energy of Bi_2_WO_6_/BiOCl nanocomposite decreased with the increasing content of Bi_2_WO_6_. These results demonstrate that a strong interface interaction between BiOCl and Bi_2_WO_6_ in the nanocomposites is effective to improve the visible light response.

### 3.2. Photocatalytic Activity Evaluation

**Figure 7:**
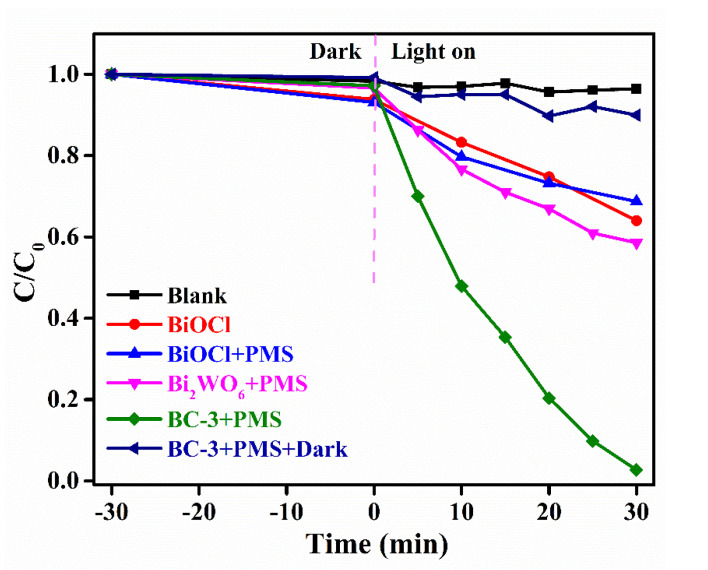
Photocatalytic properties of BPA degradation using various systems. Reaction condition: initial BPA concentration of 10 mg/L, PMS dosage of 1 mmol/L, catalyst addition of 1 g/L.

The catalytic properties of the samples were examined by BPA degradation with PMS activation. Figure 7 shows the variations in BPA concentrations under various systems. The control experiment revealed that PMS could hardly degrade the BPA molecule. The removal rates of BPA were approximately 31.1% and 41.4% by the pure BiOCl and Bi_2_WO_6_ with PMS activation under visible light irradiation, respectively. As exhibited, the removal rate of BPA reached 97.4% by BC-3 under the same condition. Obviously, the removal rate of about 10.1% demonstrates that BC-3 cannot activate the PMS molecule without light irradiation. These results suggest that BC-3 exhibits an enhancement performance in BPA degradation with PMS activation under visible light irradiation.

**Figure 8:**
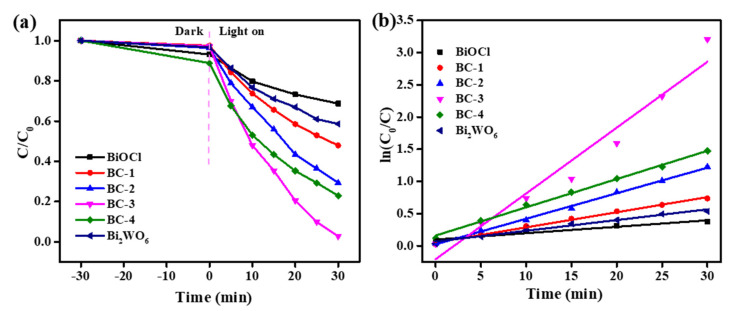
Degradation curves of BPA with PMS activation over the Bi_2_WO_6_/BiOCl nanocomposites with various contents of Bi_2_WO_6_ (**a**) and the corresponding kinetics fitting curves (**b**). Reaction condition: initial BPA concentration of 10 mg/L, PMS dosage of 1 mmol/L, catalyst addition of 1 g/L.

Furthermore, the effects of Bi_2_WO_6_ in the Bi_2_WO_6_/BiOCl nanocomposite on the BPA degradation with PMS activation were investigated, and the results are shown in Figure 8. As the content of Bi_2_WO_6_ increased from 0 to 75.0%, the removal efficiency firstly increased and then decreased. Notably, the BC-3 nanocomposite showed the highest photocatalytic activity for PMS activation. This can be attributed to the successful construction heterojunctions between the Bi_2_WO_6_ and BiOCl, accelerating the charge separation. However, the excessive Bi_2_WO_6_ might decrease the interface effect. The kinetic behavior of the samples for BPA degradation was investigated. The reaction kinetics could be fitted well by the principle of kinetics pseudo first-order reaction [44,45]. The rate constants for BPA degradation by BiOCl, Bi_2_WO_6_, BC-1, BC-2, BC-3, and BC-4 with PMS activation were 0.010, 0.017, 0.024, 0.040, 0.102, and 0.044 min^−1^, respectively. So, the BC-3 displayed better photocatalytic activity than pure BiOCl and Bi_2_WO_6_, evidencing the synergistic effect between BiOCl and Bi_2_WO_6_. These results suggest that the content of Bi_2_WO_6_ is important to optimize the catalytic activities of the nanocomposites.

**Figure 9:**
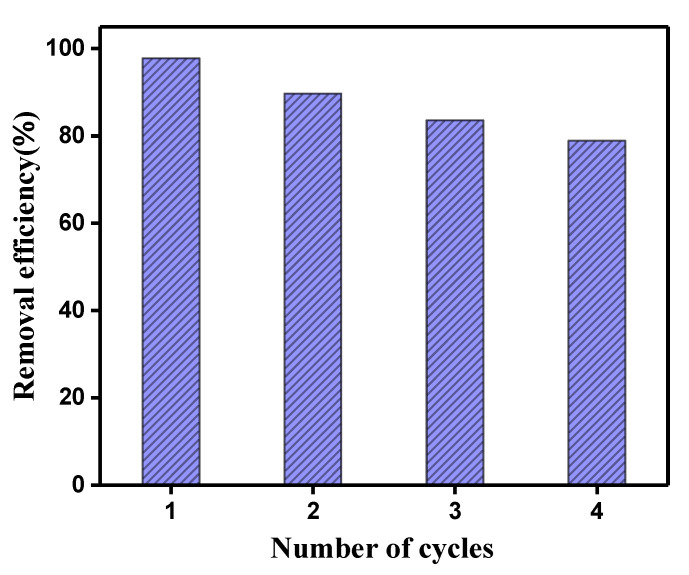
The recycling test of the prepared nanocomposite. Reaction condition: initial BPA concentration of 10 mg/L, PMS dosage of 1 mmol/L, catalyst addition of 1 g/L.

The stability and reusability of the catalysts are crucial for their practical applications [10,31]. Thus, the catalytic stability of the Bi_2_WO_6_/BiOCl nanocomposites was explored through the recycling reaction of BPA degradation. From Figure 9, a slight decrease in the catalytic activity of the BC-3 nanocomposite was observed after four cycles, illustrating that the nanocomposites have robust stability to activate PMS for BPA degradation and great potential in their practical application.

### 3.3. Photocatalytic Reaction Mechanisms

**Figure 10:**
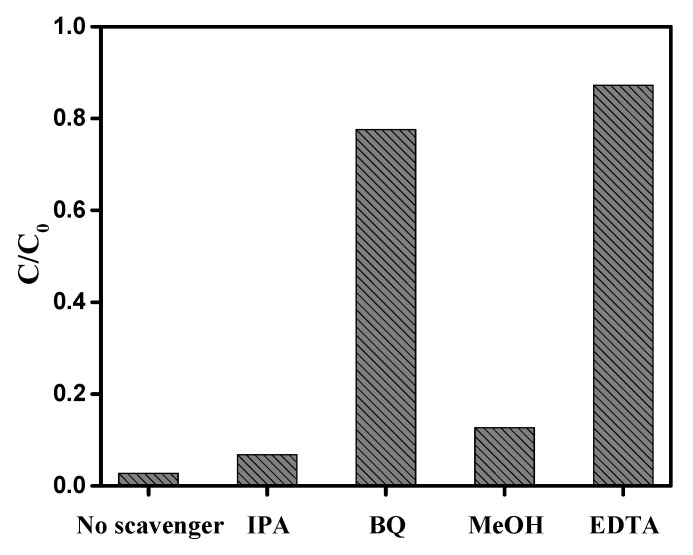
The radical trapping experiment in the PMS activation by BC-3 nanocomposite for BPA degradation. Reaction condition: initial BPA concentration of 10 mg/L, PMS dosage of 1 mmol/L, catalyst addition of 1 g/L.

To explore the underlying mechanism, the active radical species generated in the photocatalytic process were tested by radical trapping experiments with benzoquinone (BQ), methanol (MeOH), isopropanol (IPA), and ethylenediamine tetraacetic acid disodium salt (EDTA) as radical scavengers, respectively [2,6,26]. Figure 10 shows that the degradation efficiency of BPA was slightly reduced with the addition of IPA, indicating that •OH has a negligible effect on BPA degradation. Furthermore, the degradation of BPA could be significantly limited by EDTA and BQ. Therefore, the holes (*h*^+^) and superoxide radical (•O_2_^−^) play pivotal roles in BPA degradation. Furthermore, the degradation of BPA was partially limited by methanol, which could be attributed to the rapid conversion of sulfate radicals. Therefore, the *h*^+^, SO_4_^•−^, and •O_2_^−^ are important active radicals in the degradation process.

**Figure 11:**
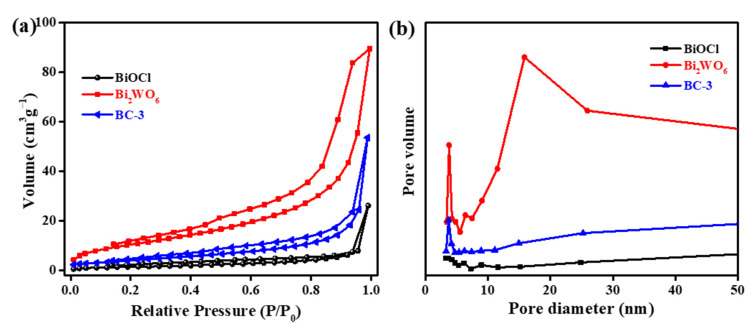
N_2_ adsorption–desorption isotherm (**a**) and pore size distribution (**b**) of pure BiOCl, Bi_2_WO_6_, and BC-3 nanocomposite.

Generally, the specific surface area is a key factor influencing the catalytic ability of the catalyst [10,19]. Hence, N_2_ adsorption–desorption measurements were taken to reveal BET surface area and pore structure. The nitrogen sorption isotherms (Figure 11a) revealed that all the samples belonged to type IV isotherms with H3 loops, illustrating mesoporous structures [31,40]. The pore size distribution curves illustrate the coexistence of abundant mesopores and macropores (Figure 11b). The BC-3 nanocomposites showed larger specific surface area than pure BiOCl (5.394 m^2^/g), but lower than pure Bi_2_WO_6_ (Table 1). Therefore, the specific surface areas and pore size distributions are not the main reason for the enhanced photocatalytic performance of the samples.

**Figure 12:**
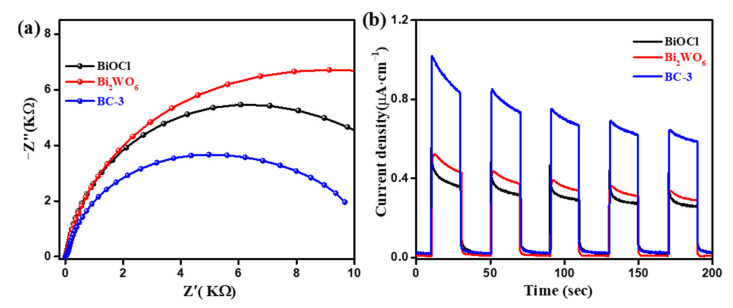
The EIS (**a**) and photocurrent response (**b**) of pure BiOCl, Bi_2_WO_6_, and Bi_2_WO_6_/BiOCl nanocomposites.

The separation efficiency and kinetic barrier of Bi_2_WO_6_/BiOCl were revealed by EIS and photocurrent response. Sensitive and reproducible photocurrent responses of the sample under on/off visible light irradiation can be observed from Figure 12a. The BC-3 nanocomposite had higher photocurrent responses than pure BiOCl and Bi_2_WO_6_, illustrating an efficient charge transmission [30,42]. Furthermore, Figure 12b shows that the arc radius of the BC-3 nanocomposite was smaller than pure BiOCl and Bi_2_WO_6_, which suggests a lower charge transfer resistance [16,31]. Thus, the heterojunction interface between BiOCl and Bi_2_WO_6_ could provide a driving force for charge separation and transfer.

**Figure 13:**
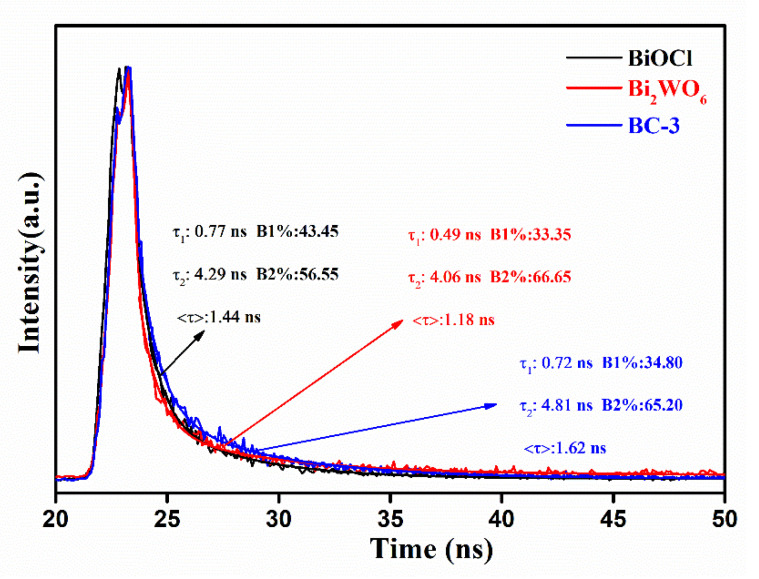
The TRPL spectra of pure BiOCl, Bi_2_WO_6_, and Bi_2_WO_6_/BiOCl nanocomposites.

The TRPL spectra was used to further identify the charge transfer dynamics. As depicted in Figure 13, the curves are fitted well based on a two exponential decay function [25,31,46]. The average PL lifetimes of pure BiOCl, Bi_2_WO_6_, and BC-3 nanocomposite were calculated to be 1.44, 1.18, and 1.62 ns, respectively. Obviously, the average lifetimes of BC-3 nanocomposites are longer than those of pure BiOCl and Bi_2_WO_6_, which further indicates the most efficient migration of photoexcited charges [25,31]. As a result, a notable synergetic effect between BiOCl and Bi_2_WO_6_ can optimize the electronic structure and suppress the recombination of charge carriers, thus promoting the catalytic performance.

**Figure 14:**
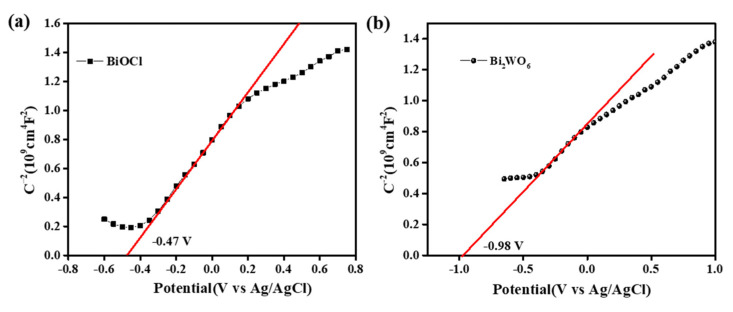
Mott–Schottky curves of the pure BiOCl and Bi_2_WO_6_.

The Mott–Schottky plots of the samples are shown in Figure 14. The flat-band potential (E_fb_) could be determined based on the Mott–Schottky curves [16,47]. The positive slope of the curves suggests that the BiOCl and Bi_2_WO_6_ are n-type semiconductor. Notably, the E_fb_ values of BiOCl and Bi_2_WO_6_ were determined to be −0.47 and −0.98 V (vs. Ag/AgCl), respectively. Therefore, the CB potential values of the BiOCl and Bi_2_WO_6_ were −0.25 and −0.76 V (vs. NHE, pH = 7), respectively [23,31]. Based on these results, the valence band (VB) positions were determined to be 3.06 and 1.90 V. These results indicate that the construction of a heterostructure interface between the BiOCl and Bi_2_WO_6_ can significantly improve photoinduced carrier separation efficiency and photocatalytic efficiency due to the matching energy levels.

**Figure 15:**
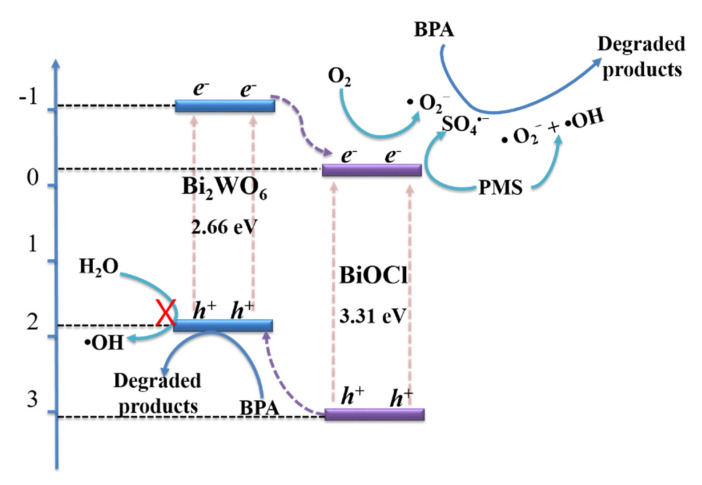
A proposed mechanism for BPA degradation via Bi_2_WO_6_/BiOCl nanocomposites with PMS activation.

Based on the above analysis, the possible mechanism for BPA degradation is portrayed in Figure 15. The heterojunction structure is obtained at the interface between BiOCl and Bi_2_WO_6_. Under light irradiation, Bi_2_WO_6_ is excited to form electrons and *h*^+^ [38,43]. The photogenerated electrons flow to the heterojunction interface, then to the CB of the BiOCl. The accumulation of electrons at the CB of the BiOCl reduce O_2_ and PMS in the solution to generate •O_2_^−^ and SO_4_^•−^ radicals [5,48]. Meanwhile, the generated *h*^+^ in the VB of BiOCl can easily accumulate at the surface of Bi_2_WO_6_ to directly degrade organic pollutants [40]. The •OH cannot be produced by *h*^+^ in the VB of Bi_2_WO_6_ due to its low oxidizing capacity, whereas a small amount of •OH is formed by the contribution of •O_2_^−^ or PMS [26,42]. This result agrees well with the reactive species trapping experiments.

## 4. Conclusions

In summary, novel Bi_2_WO_6_/BiOCl heterojunction nanocomposites were successfully designed and fabricated by a facile and effective strategy. The heterojunction was fabricated by in situ growth of Bi_2_WO_6_ on the surface of BiOCl. The Bi_2_WO_6_/BiOCl nanocomposites possess excellent performance in PMS activation for BPA degradation under visible light irradiation. The Bi_2_WO_6_/BiOCl composite with 75.0% of Bi_2_WO_6_ exhibited the optimal catalytic performance. A possible photocatalytic mechanism for BPA degradation with PMS activation over Bi_2_WO_6_/BiOCl nanocomposites was systematically investigated. The improved catalytic performance of the Bi_2_WO_6_/BiOCl is attributed to the strong interaction between the Bi_2_WO_6_ and BiOCl, resulting in an enhanced photoabsorption and a more efficient charge interfacial separation and transfer. This work might provide new inspiration for designing efficient composites for the environmental remediation of organic contaminants.

## Figures and Tables

**Table 1:** The S_BET_ and average pore size of pure BiOCl, Bi_2_WO_6_, and BC-3 nanocomposite.

Sample	S_BET_(m^2^∙g^−1^)	Average Pore Size (nm)
BiOCl	5.394	3.292
Bi_2_WO_6_	46.202	3.792
BC-3	16.129	3.704

## Data Availability

Data is contained within the article.
